# Notch pathway: a bistable inducer of biological noise?

**DOI:** 10.1186/s12964-019-0453-0

**Published:** 2019-10-22

**Authors:** Filip Vujovic, Neil Hunter, Ramin M. Farahani

**Affiliations:** 1 IDR/Westmead Institute for Medical Research, Sydney, Australia; 2 Faculty of Medicine and Health, University of Sydney, Sydney, NSW 2145 Australia

## Abstract

**Abstract:**

Notch signalling pathway is central to development of metazoans. The pathway codes a binary fate switch. Upon activation, downstream signals contribute to resolution of fate dichotomies such as proliferation/differentiation or sub-lineage differentiation outcome. There is, however, an interesting paradox in the Notch signalling pathway. Despite remarkable predictability of fate outcomes instructed by the Notch pathway, the associated transcriptome is versatile and plastic. This inconsistency suggests the presence of an interface that compiles input from the plastic transcriptome of the Notch pathway but communicates only a binary output in biological decisions. Herein, we address the interface that determines fate outcomes. We provide an alternative hypothesis for the Notch pathway as a biological master switch that operates by induction of genetic noise and bistability in order to facilitate resolution of dichotomous fate outcomes in development.

**Graphical abstract:**

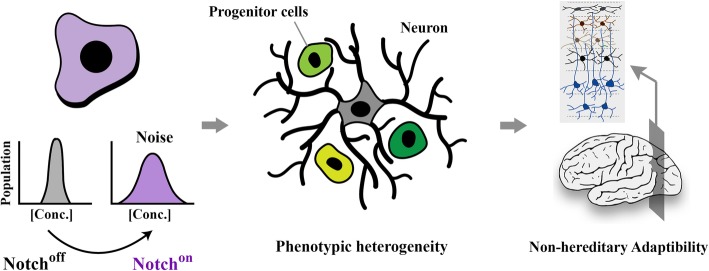

## Introduction

“*I can add colors to the chameleon, change shapes with Proteus for advantages, and set the murderous Machiavel to school.*” These are the words that Shakespeare used to describe the character of the ‘villain’ King Richard III. Yet, one would struggle to find better words to illustrate the biology of the elusive protein Notch. In metazoans, imprints of the Notch signalling pathway are evident at every level that a binary decision is made. Notch signalling regulates self-renewal of progenitor cells [1]. Yet, the same pathway activates a differentiation program in progenitor cells [2]. Further, Notch signalling directs the differentiation fate of progenitor cells. In differentiating neural crest populations, cells with increased activity of the Notch signalling pathway (Notch^high^) assume a glial fate bias as opposed to the neuronal differentiation of cells with reduced Notch signalling (Notch^low^) input [3]. Despite redundancies built into the Notch pathway [4], how could simple signalling cues communicate pleiotropic, context dependent, and sometimes opposing outcomes?

Herein, we provide evidence as the basis for an alternative interpretation of the Notch pathway as a master switch for induction and regulation of biological noise [5]. We propose that genetic noise is invoked by the Notch signalling cascade and that noise directs stochastic fate decisions by tipping the balance in favor of an outcome [6]. The proposed hypothesis could provide an explanation for the context-dependent activity of the Notch signalling cascade.

## Notch signalling pathway: an overview

Notch signalling pathway is a key evolutionary innovation of metazoans [7]. Notch is a transmembrane protein that includes an extracellular domain consisting of multiple epidermal growth factor-like (EGF) repeats, and an intracellular domain comprising ankyrin (ANK) domains, a transactivation domain and a PEST domain [8]. In canonical Notch signalling, the receptor interacts with its ligand (members of Delta-Serrate-Lag (DSL) type) on a neighbouring cell. In consequence, Notch intracellular domain (NICD) is cleaved [9] and shuttled to the nucleus. NICD can also be generated by non-canonical pathways independent of canonical ligands [10]. In addition, a truncated isoform of Notch that corresponds to NICD is directly transcribed by the activity of an alternative intragenic promoter in T-cells [11]. The topology of Notch signalling pathway is comprehensively reviewed by Andersson et al. [9]. Regardless of the mode of activation, nuclear NICD binds to the CSL family of DNA-binding proteins and activates transcription of targeted genes [12].

## Notch transcriptome: plastic and versatile

Genomic loci that are *trans*-activated downstream to Notch signalling show significant differences in various cell types and different contexts [9]. A consensus transcriptome or even an obligatory responsive gene, is yet to be identified [9]. Even downstream genes such as Hey1 and Hes5 are not consistently *trans*-activated by Notch signalling [9]. Several factors are involved in the reported versatility of the Notch transcriptome. The Notch transcription complex can bind to two distinct types of genomic response elements, monomer-binding sites and sequence-paired sites [13]. It is estimated that one third of direct Notch target genes are regulated via sequence-paired sites. Cooperative assembly of Notch transcription complexes on sequence-paired sites increases the sensitivity of Notch target genes to variation (i.e. noise) in signal strength [14]. The other important source of variability in Notch signalling is activity of super-enhancers that are in turn regulated by additional tissue-specific transcription factors [15]. Further, the observation that accessibility of Notch-responsive enhancers are regulated by chromatin remodelers [16] may in part explain the reported variability in the Notch signalling cascade. Emergence of noise would also be facilitated by *cis*-inhibition, where Notch receptors are inhibited by the ligands present within the same cell [17]. However, factors that introduce variability into Notch signalling output are not restricted to the network itself.

Activity of the Notch pathway adapts to various phases of cell cycle with peak signalling detected at M/early G1 phase [18]. Similar to Notch, the downstream targets, e.g. Hes-1, demonstrate sub-oscillations due to negative self-regulation, although at a higher frequency (≈2–3 h for Hes-1) [19]. Mechanisms that underpin the temporal variability of the Notch signalling pathway expand beyond those operating within an individual cell. This is because Notch signalling propels heterogeneity at a population level by lateral inhibition [20]. In the process of lateral inhibition, a Notch^high^ cell induces repression of the Notch receptor in the adjacent cells. These observations highlight a major hurdle in generating meaningful population-level readouts of the Notch signalling cascade. A challenge that emanates from the imprinted genetic noise in the functional topology of the Notch pathway. This behavior, however, admits an alternative interpretation. What if the induction of genetic noise is the main function of the Notch signalling cascade? Probing this possibility requires insight into the definition and the utility of noise in biological decision-making.

## Noise: a biological perspective

Although it remains hard to define with precision, genetic noise can be interpreted as random fluctuations in the intensity of a signal, leading to an altered stoichiometric relationship between the input and the output signals. The fluctuations can be instructed by extrinsic and intrinsic sources [5]. Intrinsic noise describes stochastic events that occur within an individual cell and that alter the intensity of a signal. Such stochastic events are an inherent property of transcriptional, post-transcriptional and translational dynamics [21] during generation and transmission of a biological signal. Stochastic events such as replication-transcription conflict [22] and RNA polymerase backtracking mediated by R-loop formation [23] are examples of events that invoke intrinsic noise in an individual cell. On the contrary, extrinsic noise is communicated by exogenous sources such as oscillatory cascades that regulate progression of cell cycle [24] or environmental stressors [25].

Induction or amplification of genetic noise is an important evolutionary pro-survival strategy in unicellular and multicellular organisms. This is because genetic noise fosters phenotypic heterogeneity in a population. In response to extreme environmental stressors, *Bacillus subtilis* amplifies transcriptional noise to access the program that activates competence [26]. Competence in *B. subtilis* is a transient unstable state during which exogenous DNA is internalised and integrated into the host genome. The unstable state is transiently invoked by stressor-mediated genetic noise and after revision of the genome, competent cells return to the default non-competent state [27]. Similarly, spatial patterning of the retinal color-vision mosaic during development of *Drosophila melanogaster* is instructed by stochastic transcriptional fluctuations (transcriptional noise) that bring about probabilistic resolution of dichotomous fate outcomes [28]. The amplification of biological noise during differentiation is required to introduce instability into the steady-state molecular profile of a progenitor cell and to invoke bistability as a conseuqence [6]. In the latter example, key events that instruct noise-dependent specification of differentiation fates in the ommatidium of the *Drosophila* eye are regulated by the Notch signalling cascade [29]. This finding is not surprising as the Notch signalling pathway interfaces with biological noise at various levels.

## Does notch instruct genetic noise at an individual cell level?

Notch pathway, upon activation, boosts global transcriptional activity by multiple parallel mechanisms. In the absence of NICD, its binding partner, recombination signal binding protein-J (RBP-J), recruits various co-repressors and inhibits gene expression [30]. Binding of NICD to RBPJ and subsequent recruitment of Mastermind Like Transcriptional Coactivator (MAML) to this complex terminates repression and activates transcription [4]. This increased gene expression is supported by NICD/RBPJ-mediated activation of enhancers that improve processivity of RNA polymerase-II (RNAP-II) [15]. Therefore, the primary outcome of Notch signalling is a global amplification of gene expression that is mediated by direct activity of NICD. However, some Notch responsive genes are transcriptional repressors that reshape the transcriptome in a subsequent phase. Here, it is proposed that the clash of primary NICD-mediated transcriptional activation and secondary transcriptional repression facilitates the generation and amplification of biological noise.

**Fig. 1:**
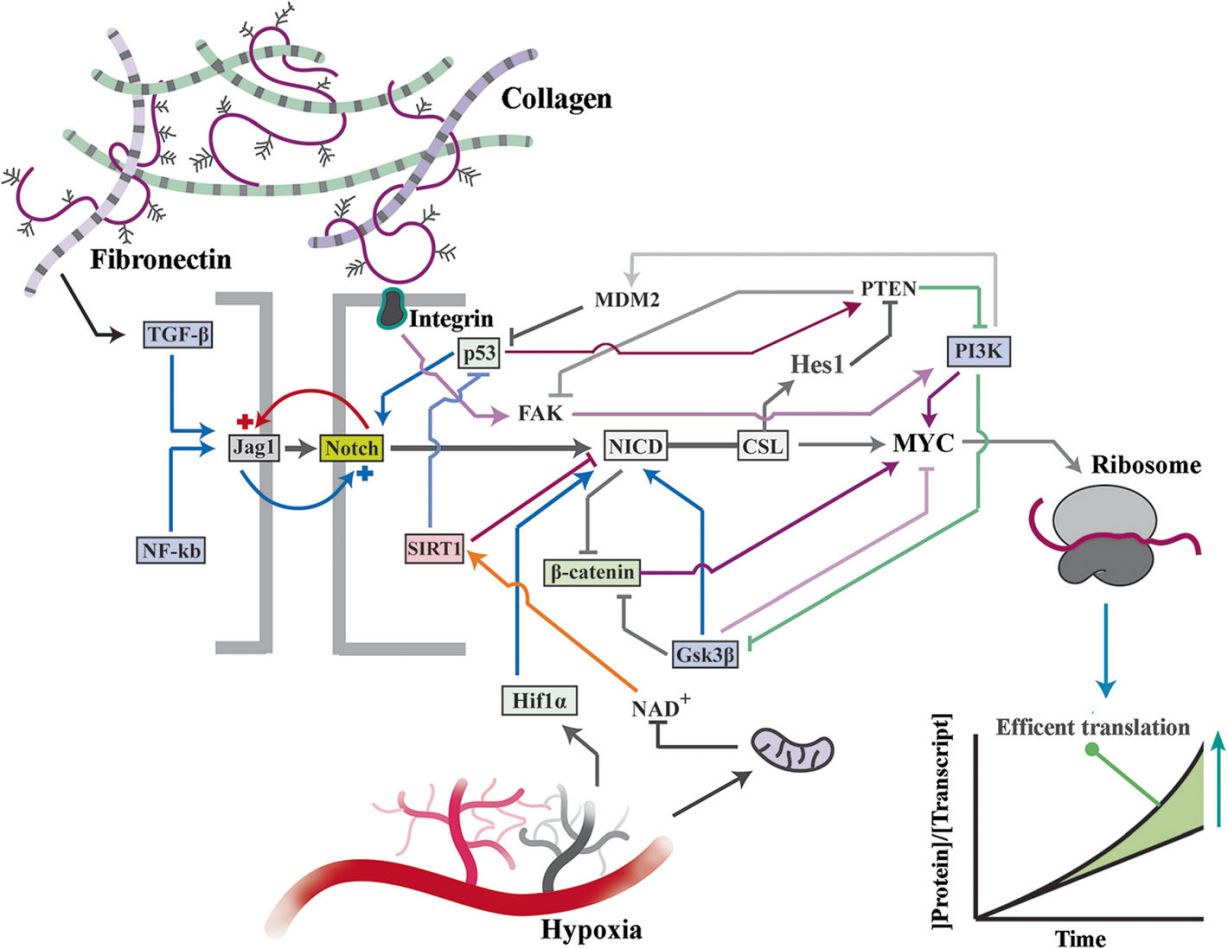
Notch pathway amplifies intrinsic noise by interactions that improve the efficiency of global protein synthesis. Schematic image shows interaction of a sender (Jag^+^) and a receiver (Notch^+^) and downstream molecular events instructed by these interactions. System-level interactions of Notch pathway lead to enhanced availability of Myc. Global amplification of protein synthesis following upregulated activity of Myc amplifies noise by distorting the stoichiometric relationship between transcripts and associated proteins

As mentioned previously, a major source of genetic noise is altered stoichiometric relationship between transcriptional input and translational output. Such discrepancy is generally induced by enhanced capacity of cells for translation of available transcripts [31]. There is evidence that the Notch signalling cascade plays a major role in regulating the translational capacity of cells by *trans*-activating the c-Myc gene (Fig. 1) [32, 33]. Myc proto-oncogene is a key pro-anabolic regulator that enhances growth and proliferation rate of cycling cells [34]. In order to support pro-anabolic activity of cycling cells, Myc drives ribosome biogenesis and enhances global protein synthesis during G1 phase of cell cycle [35]. This occurs by RNAPI-mediated transcription of 18S, 5.8S and 28S rRNAs [36], and also RNAPII-mediated transcription of ribosomal proteins [37] together with up-regulation of translation initiation factors [38]. It is further proposed the activation of Myc downstream to Notch signalling could improve global translational activity by a second mechanism that involves the mTOR (Target of Rapamycin) pathway. Notch signals provide positive input into the mTOR cascade by upregulating Myc activity [39]. Signals communicated by the mTOR pathway inhibit autophagy [40] and positively regulate ribosome biogenesis and global protein synthesis by enhancing transcription of rRNA genes [41]. Given that ≈60% of the total capacity of the transcriptional machinery is dedicated to ribosome biogenesis in G1 phase of cell cycle [42, 43], one would expect a parallel reduction in transcriptional output from other genomic loci in this phase of cycle. Inefficient transcription combined with amplified translational capacity could result in distorted transmission of biological signals and induction of genetic noise [44, 45]. Arguably, the impact of Notch-induced noise will be proportional to the length of G1 phase of cell cycle that accommodates active Myc signalling. The PI3K/Akt [46] and Wnt/β-Catenin [47] are major cascades that accelerate progression through G1 phase and hence restrict translation-related noise. Remarkably, Notch signalling critically interfaces with botch cascades to amplify noise by lengthening G1 phase.

## Altered network topology of the PI3K/Akt pathway in Notch^on^ cells

**Fig. 2:**
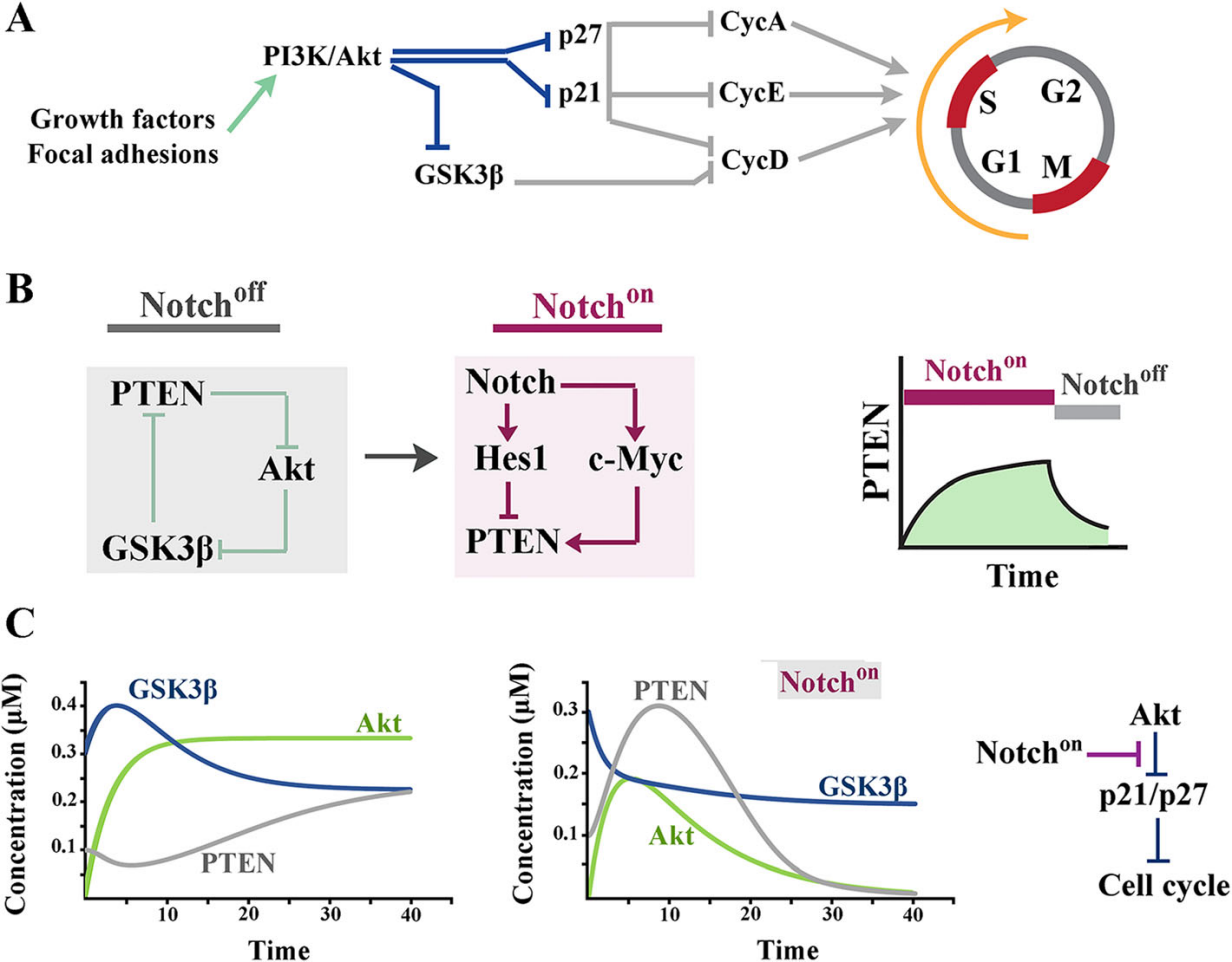
Altered topology of the PI3K/Akt pathway in Notch^on^ cells. **a** The schematic diagram demonstrates inhibitory interactions of the PI3K/Akt cascade with major downstream suppressors of cell cycle progression. **b** Network topology of a simple negative feedback loop formed by PTEN/GSK3β/Akt in the Notch^off^ state (left graph) and subsequent to activation of Notch signalling (Notch^on^, right graph). The right graph shows potential impact of incorporating PTEN in I1-FFL loop in the Notch^on^ state. **c** Modeling demonstrates time course of equilibration of Akt signalling subsequent to activation of the cascade by a single exposure to growth factors in the Notch^off^ (left graph) and the Notch^on^ (right graph) states. Kinetic biochemical modeling was accomplished using VCell software. Schematic diagram (left) shows suppression of the Akt-mediated inhibition of p21 and p27 in the Notch^on^ state

Upon activation of notch signalling, NICD integrates into and alters signalling topology of the PI3K/Akt pathway (Fig. 2a, b). In Notch^off^ cells, a tripartite negative regulatory loop that comprises PTEN (phosphatase and tensin homologue deleted on chromosome 10), GSK3β, and PI3K cascade regulates activity of downstream Akt kinase (Fig. 2b). In Notch^off^ state, PTEN is phosphorylated by GSK3β on Thr-366 leading to reduced stability of the former protein [48]. Reduced stability of PTEN could stimulate PI3K signalling output as PTEN inhibits the latter pathway by dephosphorylating phosphoinositides [49]. Enhanced PI3K signalling activity, in turn, leads to Akt-mediated phosphorylation and inhibition of GSK3β [50]. This tripartite feedback loop could regulate signalling capacity of Akt subsequent to its activation by mitogens, e.g. growth factors (Fig. 2c). Interestingly, the topology of this loop is radically reconfigured in Notch^on^ state.

The Notch downstream mediator, Hes-1, represses transcription of PTEN in Notch^on^ state [51]. In parallel, upregulation of MYC by Notch [32, 33] *trans*-activates PTEN [52]. This altered topology is consistent with an incoherent type-I feedforward loop (I1-FFL) known to be an effective pulse generator [53]. As such, kinetic modelling using VCell predicted that integration of PTEN into the I1-FFL in the notch^on^ state would trigger an initial activation (PTEN^high^ phase) followed by subsequent GSK3β-mediated repression (PTEN^low^ phase) of its phosphatase activity (Fig. 2b). The PTEN pulse in the notch^on^ state would exert a strong repressive impact on Akt leading to premature termination of the PI3K/Akt signalling activity. Restricted activity of Akt signalling in notch^on^ state could potentially decelerate progression of cell cycle [46]. This is because Akt-dependent phosphorylation of p21 at Thr-145 is required to abolish the inhibitory impact of p21 on DNA replication [54]. In agreement with our modeling results (Fig. 2c), it was recently demonstrated that PTEN mediates Notch-dependent cell cycle arrest during angiogenesis [55].

## Altered activity of the Wnt/β-catenin pathway in Notch^on^ cells

Downstream to activated Wnt/ signalling, β-catenin translocates into the nucleus where it binds to the *trans*-activation partner, TCF3/LEF1 [56] and upon which transcription is initiated [57]. Two major drivers of G1 phase of cell cycle, cyclin-D1 [47] and c-Myc [58], are amongst those genomic loci *trans*-activated by nuclear β-catenin. It is also known that β-catenin can suppress p53 acetylation and transcriptional activity [59]. Downregulation of p53 signalling desensitizes G1/S checkpoint [60, 61] leading to accelerated transitioning into S phase of cell cycle. It is, therefore, expected that mechanisms that enhance Wnt/β-catenin signalling would also support accelerated navigation though short G1 phase [62].

**Fig. 3:**
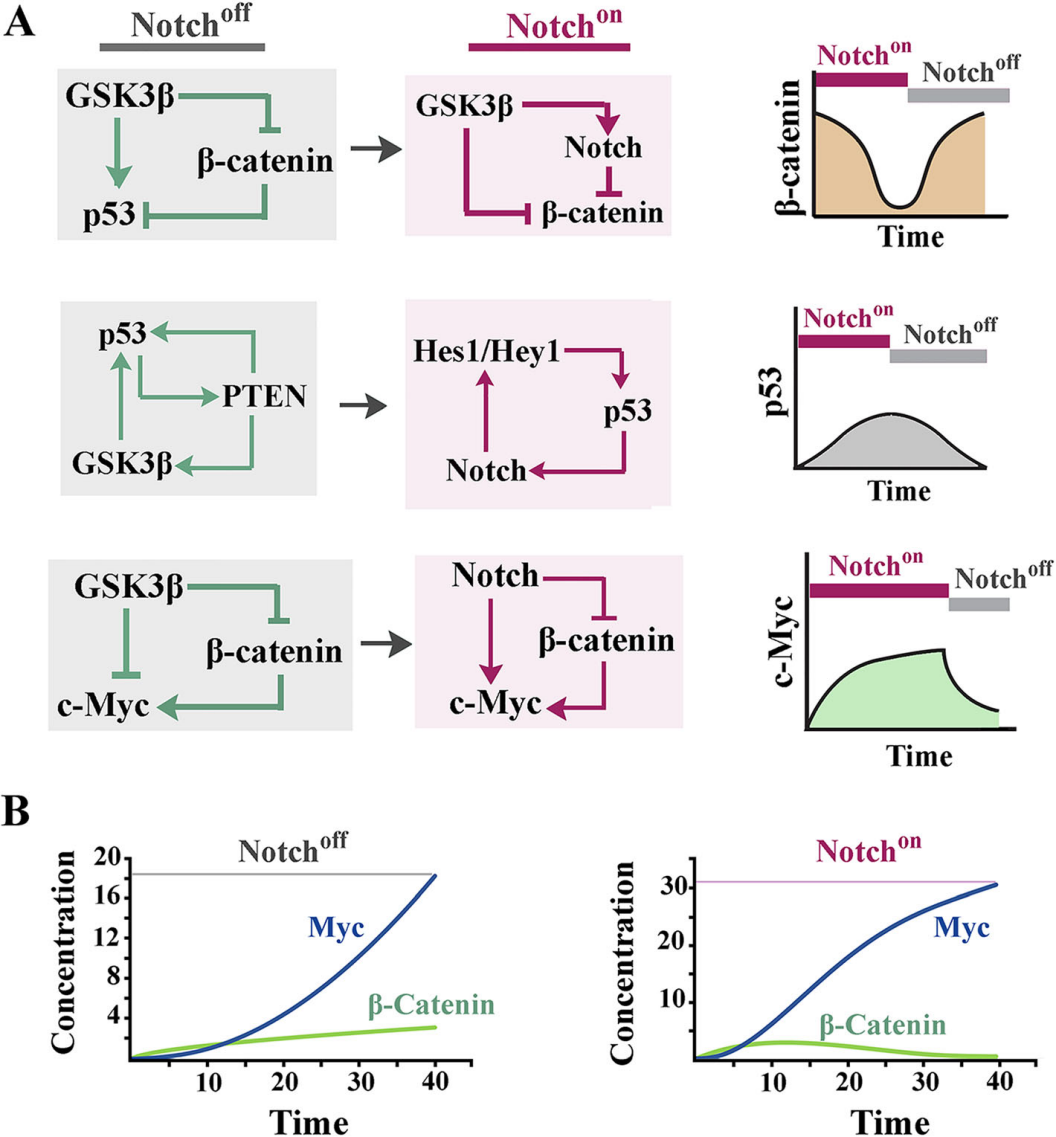
Altered topology of the Wnt/β-catenin pathway in Notch^on^ cells. **a** Left panels show network topology of simple feedback loops that regulate signalling output of the Wnt/β-catenin pathway in the Notch^off^ and the Notch^on^ states. Line diagrams (right) show predicted impact of altered network topology the Notch^on^ state on cytoplasmic availability of β-catenin, p53, and Myc. **b** Modeling demonstrates time course of equilibration of Myc and β-catenin subsequent to activation in the Notch^off^ and the Notch^on^ states. Kinetic biochemical modeling was accomplished using VCell software

Notch signalling antagonizes the Wnt/β-catenin pathway by multiple direct and indirect interactions (Fig. 3a) [63]. It is reported that Notch can physically associate with active β-catenin and downregulates the latter protein [64]. In addition to direct interaction, downregulation of PI3K/Akt via Notch/PTEN axis [55] (see previous section) could potentially reduce the half-life of β-catenin. This is because free cytoplasmic β-catenin is tightly regulated by a destruction complex that recruits the proteins and degrades it subsequent to phosphorylation by Gsk-3β [65]. Inhibition of Akt, a negative regulator of GSK3β [50], would potentially boost phosphorylation and degradation of β-catenin by Gsk-3β. Further to inhibition of Gsk3β, Akt phosphorylates β-catenin at Ser-552 and causes its disassociation from junctional complexes followed by shuttling into the nucleus to activate gene expression [66]. Therefore, input from Notch/PTEN axis [55] in the Notch^on^ state would downregulate the Wnt/β-catenin cascade in parallel to inhibition of the PI3K/Akt pathway. Consistent with this proposal, biochemical modeling of β-catenin/Myc axis predicted accelerated downregulation of both proteins in the Notch^on^ state (Fig. 3b). Remarkably, the cross-talk between Notch signalling and cell cycle does not only via modulation of the cascade topology of the PI3K/Akt and the Wnt/β-catenin pathways. Notch signalling orchestrates transition from G1 to S phase of cell cycle by multiple other interactions that target the G1/S checkpoint machinery.

## Notch signalling input sensitizes G1/S checkpoint

In the Notch^on^ state, signalling activity of p53 is enhanced by Hey1-mediated inhibition of the key p53 inhibitor, MDM2 [67]. Stabilization of p53 enhances destruction of CDC6 (Cell Division Cycle 6) by anaphase-promoting complex [68]. CDC6 together with Cdt1 is required for loading of the minichromosome maintenance (MCM) complex in G1 phase of cell cycle and subsequent replication of DNA in S phase [69]. These proteins are components of the pre-replicative complex (pre-RC) that is formed in early G1 in anticipation of DNA replication [70]. Notch signalling also delays entry into S phase by downregulation of Minichromosome maintenance (MCM) proteins, MCM-2 and MCM-6 [71]. As a consequence, assembly of DNA pre-replication complex and progression of cycling cells into S phase will be delayed in the Notch^on^ state. Consistent with this notion, sustained expression of Notch triggers quiescence in cycling cells [2]. To this end, one may question what triggers progression of Notch^on^ G1-arrested cells into S phase? Self-limiting nature of Notch signalling and de-repression of cell cycle mediated by Notch downstream mediators are potential mechanisms that can propel entry into cell cycle of G1-arrested cells.

Hes1/5 and Hey1/2 are the key downstream mediators of Notch signalling are transcriptional repressors that bind preferentially to the canonical E-box sequence 5′-CACGTG-3′ [72]. Hes-1 propels progression of cell cycle by transcriptional repression of the cyclin-dependent kinase inhibitor, p27(Kip1) [73]. In the absence of Hes1, levels of cyclin-dependent kinase inhibitors, p27 and p57, increase significantly leading to impaired progression of cell cycle [74] and precocious differentiation [75]. Similarly, Hey-1 is known to be an effective repressor of p57 [76]. Therefore, the second wave of global transcriptional remodeling mediated by Notch downstream mediators, Hes-1 and Hey-1, facilitates progression of cycling cells into S phase. Notch signalling, on the other hand, is terminated by an auto-regulatory mechanism. Notch is degraded by autophagy, via uptake into ATG16L1-positive autophagosomes [77]. Enhanced autophagy triggers depletion of NICD and rapid progression through interphase [62]. Sustained Notch signalling requires activation of mTOR (mammalian target of rapamycin) pathway with resultant inhibition of autophagy [78]. This is because signalling by mTOR inhibits ATG1 [79] and hence abolishes initiation of autophagy [80].

**Fig. 4:**
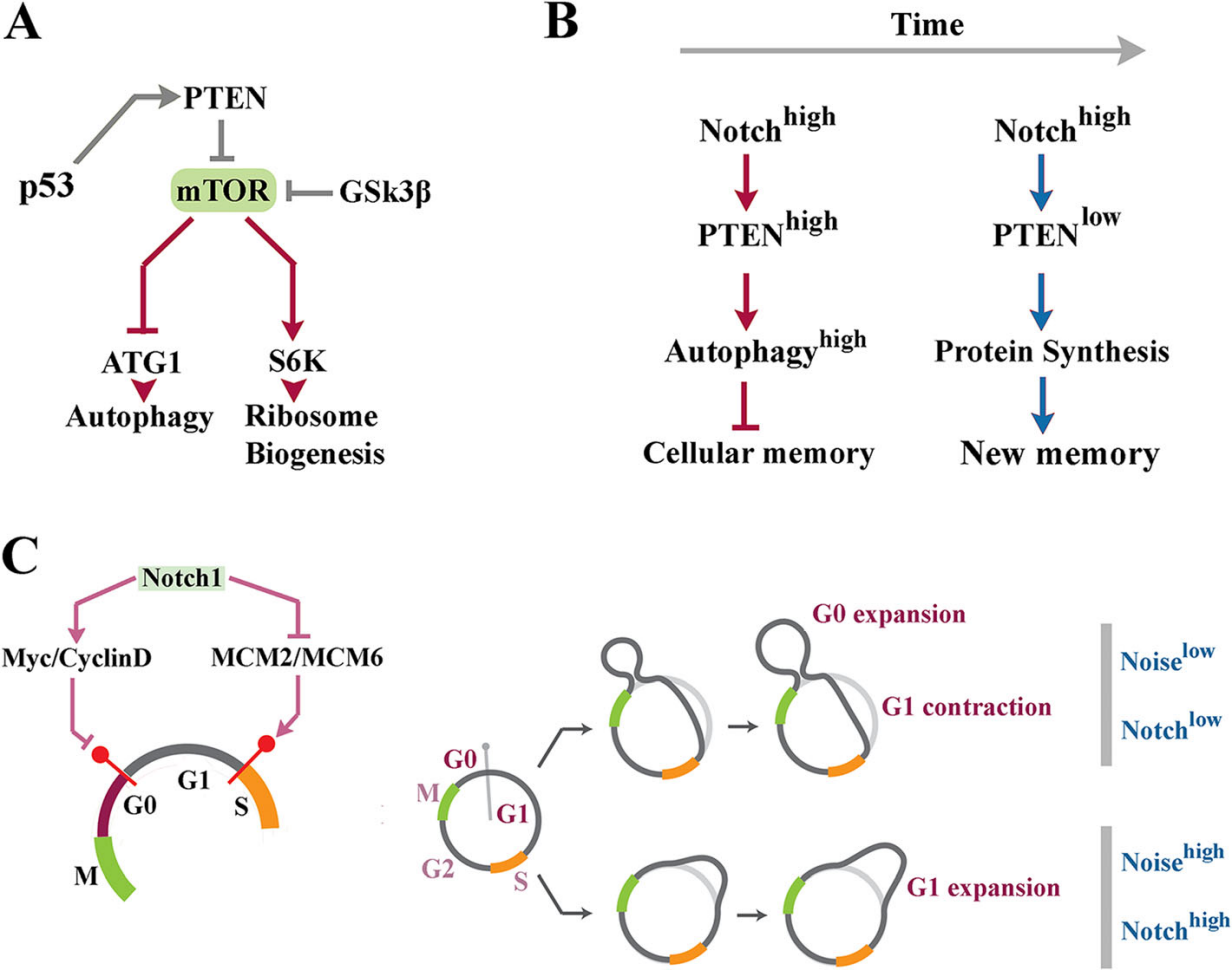
Notch-induced autophagy and translational noise as destabilizers of cellular memory. **a** The schematic diagram demonstrates molecular interactions that regulate transitioning between pro-anabolic and pro-catabolic states in a cell. **b** Subsequent to activation of Notch signalling, the PTEN^high^ and PTEN^low^ temporal windows (corresponding to the Notch^on^ state of Fig. 2c) accommodate pro-catabolic (high autophagy) and pro-anabolic (high protein synthesis) phases. **c** Notch signals facilitate G0/G1 transition by upregulating Myc and cyclin D1. On the other hand, Notch signals inhibit G1/S transition. In consequence, cycling cells dwell longer in noisy G1 characterized by amplified protein synthesis

In notch^on^ state, upregulation of PTEN that acts as a negative regulator of mTOR signalling pathway [81] could potentially enhance the autophagic flux (Fig. 4a, b). Akt, on the other hand, activates mTOR by destabilizing TSC1 (Tuberous sclerosis 1) via phosphorylation [82]. Reduced Akt signalling in the notch^on^ state could contribute to repression of mTOR and amplification of autophagy (Fig. 4a). Induction of autophagy as a consequence of Notch signalling [83] would lead to a self-limiting loop whereby amplified autophagic flux eliminates NICD [62] and facilitates progression into S phase. This mechanism is reminiscent of the self-regulatory loop that represses the p53 signalling subsequent to induction of autophagy by this protein [84]. Induction of autophagy, however, would have additional consequences for the Notch^on^ cell.

## Notch-induced noise: a destabilizer of cellular memory?

Cellular memory can be operationally defined as the repertoire of proteins that instruct and stabilize the functional state of a cell. The autophagy-mediated depletion of cytoplasmic protein repertoire is an essential transitional step in cellular reprogramming during development [85]. The most remarkable example of such activity is observed in reprogramming of highly differentiated oocyte into undifferentiated zygote by complete degradation of maternal proteins [86]. Similarly, during somatic cell reprogramming inhibition of mTORC1 and induction of autophagy facilitate transitioning into the early stochastic phase of reprogramming [87]. It is, therefore, proposed that Notch signalling amplifies genetic noise by two parallel mechanisms. Induction of autophagy partially erases the cellular memory by degradation of existing proteins (Fig. 4b). Simultaneously, Notch signalling increases translation efficiency by stimulating progression of cycling cells into G1 phase [47, 88] characterized by efficient ribosome biogenesis (Fig. 4b, c). We propose that increased translational noise in Notch^on^ state combined with autophagy-mediated destabilisation of cellular memory could induce bistability prior to cellular decision making [6]. The proposed role of Notch signalling in destabilizing cellular memory is bolstered by observation that differentiated quiescent cardiomyocytes reenter cell cycle following activation of Notch pathway [89].

Notch-mediated induction of autophagy and translational noise could also explain the reported versatility of Notch transcriptome [9]. Both autophagy and translational noise enhance temporal stochasticity of the Notch transcriptome by complementary mechanisms. Given the intimate association of autophagy and genetic noise with stressors [90, 91], Notch-induced noise can be regulated by exogenous cues. Such context-dependent (pleiotropic) induction of Notch-mediated noise and bistability is critical for self-organisation [92] during morphogenesis [93] and reprogramming of cells [35] during regeneration.

## Context-dependent induction of noise by Notch signalling

In mammalian cells, exogenous stressors are gated to Gsk-3β [94], p53 [95], and NF-kb [96] pathways (Fig. 1a). In this representation, integration of extrinsic stressors such as nutrient deprivation [97] and hypoxia [98] leads to activation of Gsk-3β. Phosphorylation by Gsk3β boosts Notch signalling by reducing proteasomal degradation of the latter protein [99]. Likewise, genotoxic stressors, by stabilization of p53 protein [95], would act to stimulate transcription from the notch locus [100]. Another major stressor, oxidative damage, is sensed by the NF-kb signalling pathway. Notch-1 and NF-kb communicate in a positive feedback loop. NF-kb enhances transcription of the Notch ligand Jagged-1 [101], and the downstream targets deltex-1 and hes-5 [102]. It is proposed that Notch, on the other hand, enhances nuclear retention and signalling of NF-kb by a physical interaction with the latter protein [103]. Hence, exogenous stressors that amplify Notch signalling are in turn amplified by Notch pathway. This is analogous to stressor-mediated amplification of genetic noise in *B. subtilis* albeit with a major difference. In *B. subtilis,* noise is regulated only at an individual cell level. Notch, however, controls population-level dynamics through the canonical signalling pathway.

## Notch amplifies genetic noise at a population level

**Fig. 5:**
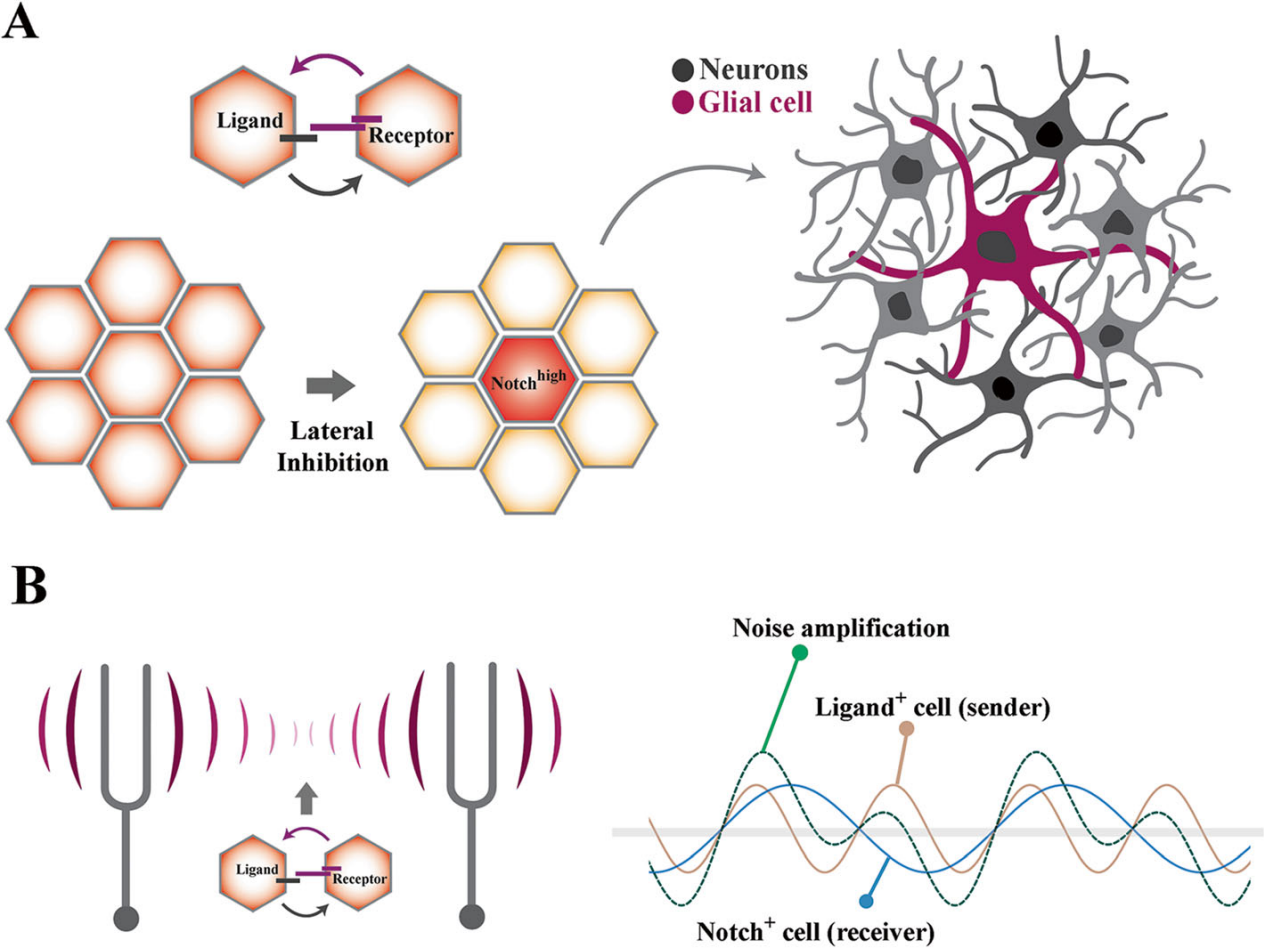
Integration of intercellular cues amplifies intrinsic noise in the recipient cell. **a** By lateral inhibition, the interacting cells express Notch ligand and receptors in a mutually exclusive manner. Such polarized expression of the ligands and receptors maximizes induction of noise in the recipient cell. Phenotypically, polarized expression of the ligand and receptors becomes manifest as optimized spatial distribution of differentiation fates associated with Notch^high^ and Notch^low^ state (e.g. glia versus neuron in development of neurosensory apparatus). **b** The intercellular cues provided by neighboring cells amplify noise-inducing activity of notch pathway in a manner akin to harmonic interaction of tuning forks. By this mechanism, every interaction that liberates NICD provides positive feedback into the recipient cell and increases the magnitude and duration of pre-existing noisy state (right schematic image where noise is represented in wave-form)

Activation of Notch by canonical ligands requires expression of these ligands by adjacent cells (Fig. 5) [4]. Notably, Notch^high^ cells stimulate expression of the Notch ligand Jagged-1 in adjacent cells via a process of lateral induction [104, 105]. It then follows that the “noisy state” of Notch^high^ cells could be further escalated by positive feedback provided by Jagged-1^high^ on adjacent cells (Fig. 5). In addition to induction of noise at an individual cell level, Notch integrates signals from other cells in a population to maximise biological noise in Notch^high^ cells. Arguably, the perpetual amplification of noise in a Notch^high^ cell could drive the cell towards the tipping point prior to resolution of a binary fate outcome.

## Notch and the trio of fate dichotomy, noise and bistability

**Fig. 6:**
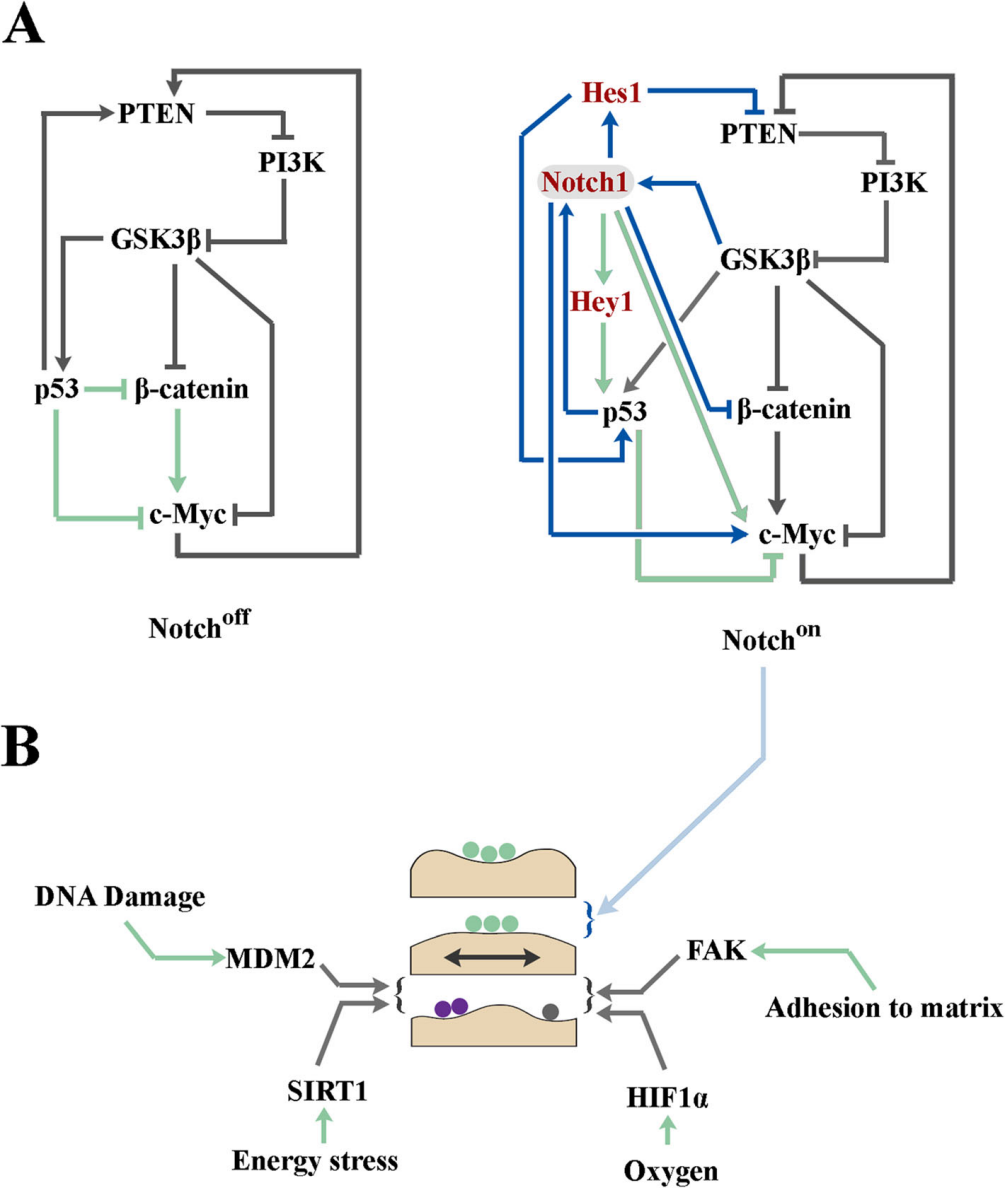
Noise-induced bistability and resolution of fate dichotomies. **a** Integrated view of global network topology of Wnt/PI3K/Myc axis following integration of the Notch signaling pathway. **b** It is proposed that Notch signaling input combines enhanced autophagic flux with amplifications of translational noise in order to destabilize the existing cellular memory (i.e. destabilize thermodynamic stability) and to invoke bistability prior to biological decision-making. Resolution of such bistability requires integration of exogenous cues that amplify or terminate the Notch signalling pathway

Fate dichotomies are a recurring theme of multicellular self-organisation during developmental morphogenesis. Resolution of such dichotomies is the ultimate outcome of a competition between a thermodynamically favoured fate and one that is repressed by the steady-state molecular dynamics of a cell and the cues provided by its surrounding milieu. Proliferation/differentiation dichotomy exemplifies a major developmental bifurcation that is iteratively resolved in cycling cells during development. In the presence of growth stimulatory factors (e.g. growth factor), two parallel fates are in direct competition; the thermodynamically favoured fate (i.e. proliferation) is the dominant outcome and quiescence or differentiation are outcomes that are repressed by signalling cues from growth factors. Such repression is a direct consequence of monostability [106] directed by stable topology of biochemical networks that function in a cell (Fig. 6a). We propose that Notch-induced noise causes a transition from monostability to bistability [107] by combined activity of autophagy (that erases cellular memory [85–87]) and translational noise (that prevents reestablishment of steady-state dynamics) (Fig. 6b). In support of our hypothesis, it has been reported that ribosome level and translational efficiency are linked to a binary threshold that controls presence or absence of selected proteins that are involved in directing the differentiation fate of cycling progenitor cells [108, 109]. In the proposed model, Notch-induced biological noise and the resultant bistability is suggested to tip the balance in favor of thermodynamically less favored outcome (e.g. differentiation in the presence of growth stimulatory factors). This explanation is consistent with the observation that amplified Notch signalling hinders progression through cell cycle while attenuated signalling stimulates proliferation of progenitor cells [2].

## Evolutionary remark

Detailed appraisal of the molecular evolution of the Notch signalling pathway is beyond the scope of the current hypothesis. However, a brief overview of the evolutionary trajectory of Notch protein provides additional insight into its function as a regulator of biological noise. As mentioned previously, Notch protein consists of multiple epidermal growth factor-like repeats, ankyrin domains, and a transactivation domain. The ankyrin domains of Notch are critical for its interactions with other partners and in particular, the CSL family of DNA-binding proteins [110, 111]. From an evolutionary perspective, ankyrin domains are highly conserved structural motifs that are found even in unicellular eukaryotes such as *Saccharomyces cerevisiae* and *Schizosaccharomyces pombe* [112]. Swi6 is a key regulatory protein in yeast that contains ankyrin domains [112] and coordinates G1/S transition [113] similar to the role of Notch proteins in regulation of the metazoan cell cycle. As such, it can be argued that the ancestral role of Notch family proteins was regulation of the cell cycle dynamics. This could have been achieved by integration of environmental cues that provided a dormant capacity for Notch to operate as a “master inducer” of noise, a function that became operational subsequent to evolution of the Notch signalling cascade in metazoan cells [7].

## The prospect for experimental validation

In order to advance the proposed hypothesis, multiple aspects of the Notch signalling pathway need to be carefully dissected. To this end, a major experimental challenge is intimate coupling of Notch signalling and autophagy. As discussed in a previous section, autophagy plays a key role in regulating half-life of NICD [77] and is in turn regulated by Notch signalling [83]. Autophagy, however, receives feedback from various other pathways that supress or stimulate the formation and maturation of autophagosomes [114]. Temporal landscape of autophagic flux is also intimately coupled to cell cycle dynamics [115]. Therefore, experimental conditions need to be standardised with respect to environmental cues and the cycling population should be synchronised with respect to cell cycle. Given the association of autophagy and Notch signalling, conventional methods of cell cycle synchronisation, e.g. serum starvation, that trigger autophagic flux are less useful for this purpose. Alternative methods of cell cycle synchronisation, such as “baby machine” [116], could be more reliable in order to study Notch signalling. Apart from experimental difficulties of investigating Notch signalling in an unbiased manner, requirement for an operational definition of genetic noise that is amenable to quantification poses a second challenge. Direct profiling of the activity of PTEN/Akt/GSK3β axis could provide insight into the level of noise. This method, however, will be confounded by inherent heterogeneity of the multicellular population. On the other hand, cell cycle landscape of a multicellular population, as an indirect but reliable proxy for the level of noise, can be accurately quantified using non-invasive methods described elsewhere [62].

## Concluding remarks

It may be argued that Notch pathway is a master switch that induces and amplifies biological noise in metazoan cells. The proposed role as an inducer of transcriptional noise could oppose the role of Wnt/β-catenin in filtering noise [117, 118], thereby providing an alternative framework for interpretation of the antagonistic interface of the Notch and the Wnt signalling pathways. In this model, noise-induced bi-stability prior to biological decision-making is suggested to be the direct outcome of the Notch signalling pathway that acts to destabilize the self-organisation signature of the Wnt/β-catenin cascade [119] in order to distort imprinted morphological traits and enhance phenotypic plasticity during development of metazoan animals. The framework proposed reconciles versatility of the transcriptome and the predictability of outcomes instructed by Notch signalling.

## Data Availability

Not applicable.

## Abbreviations

ANK: Ankyrin
DSL: Delta-Serrate-Lag
EGF: Epidermal growth factor
I1-FFL: Incoherent type-I feedforward loop
MAML: Mastermind Like Transcriptional Coactivator
MCM: Minichromosome maintenance
mTOR: mechanistic Target of Rapamycin
NICD: Notch Intracellular domain
Pre-RC: Pre-replicative complex
RBP-J: Recombination signal binding protein-J
RNAP-II: RNA polymerase-II
rRNA: ribosomal RNA
